# Bis(isonicotinamide-κ*N* ^1^)bis­[4-(methyl­amino)benzoato]zinc(II) monohydrate

**DOI:** 10.1107/S1600536809041208

**Published:** 2009-10-17

**Authors:** Tuncer Hökelek, Hakan Dal, Barış Tercan, Özgür Aybirdi, Hacali Necefoğlu

**Affiliations:** a Department of Physics, Hacettepe University, 06800 Beytepe, Ankara, Turkey; b Department of Chemistry, Faculty of Science, Anadolu University, 26470 Yenibağlar, Eskişehir, Turkey; c Department of Physics, Karabük University, 78050, Karabük, Turkey; d Department of Chemistry, Kafkas University, 63100 Kars, Turkey

## Abstract

In the title Zn^II^ complex, [Zn(C_8_H_8_NO_2_)_2_(C_6_H_6_N_2_O)_2_]·H_2_O, the Zn atom is coordinated by two 4-methyl­amino­benzoate (MAB) and two isonicotinamide (INA) ligands in a distorted trigonal-bipyramidal geometry; one of the MAB ions acts as a bidentate ligand while the other MAB and the two INA are monodentate ligands. The dihedral angles between the carboxyl groups and the adjacent benzene rings are 8.52 (22) and 5.10 (14)°. In the crystal, inter­molecular O—H⋯O and N—H⋯O hydrogen bonding links the mol­ecules into a supra­molecular structure. Weak inter­molecular C—H⋯O inter­actions are also present.

## Related literature

For niacin, see: Krishnamachari (1974 ▶) and for the nicotinic acid derivative *N*,*N*-diethyl­nicotinamide, see: Bigoli *et al.* (1972 ▶). For related structures, see: Greenaway *et al.* (1984 ▶); Hökelek & Necefoğlu (1996 ▶); Hökelek *et al.* (2009 ▶).
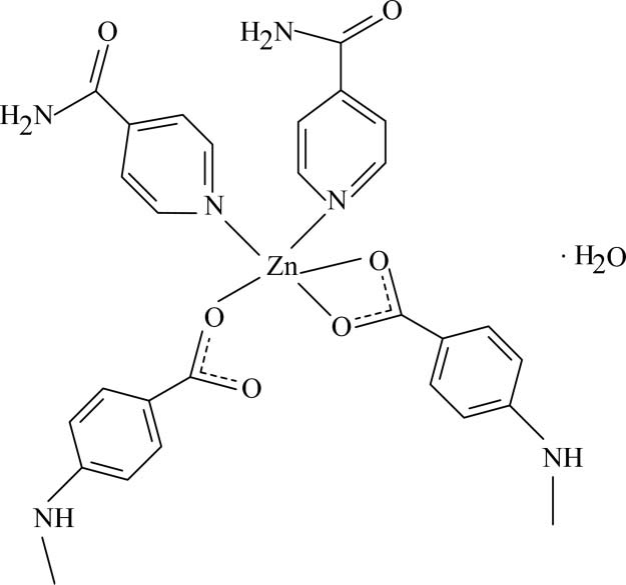


## Experimental

### 

#### Crystal data

[Zn(C_8_H_8_NO_2_)_2_(C_6_H_6_N_2_O)_2_]·H_2_O
*M* *_r_* = 627.95
Monoclinic, 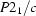

*a* = 8.1323 (8) Å
*b* = 13.2098 (12) Å
*c* = 27.219 (3) Å
β = 96.949 (6)°
*V* = 2902.6 (5) Å^3^
*Z* = 4
Mo *K*α radiation
μ = 0.90 mm^−1^
*T* = 294 K
0.55 × 0.45 × 0.35 mm

#### Data collection

Bruker Kappa APEXII CCD area-detector diffractometer
Absorption correction: multi-scan (*SADABS*; Bruker, 2005 ▶) *T*_min_ = 0.618, *T*_max_ = 0.725
27836 measured reflections
7249 independent reflections
5243 reflections with *I* > 2σ(*I*)
*R*_int_ = 0.047

#### Refinement

*R*[*F* ^2^ > 2σ(*F* ^2^)] = 0.042
*wR*(*F* ^2^) = 0.110
*S* = 1.03
7249 reflections
389 parameters
3 restraints
H atoms treated by a mixture of independent and constrained refinement
Δρ_max_ = 0.62 e Å^−3^
Δρ_min_ = −0.32 e Å^−3^

### 

Data collection: *APEX2* (Bruker, 2007 ▶); cell refinement: *SAINT* (Bruker, 2007 ▶); data reduction: *SAINT*; program(s) used to solve structure: *SHELXS97* (Sheldrick, 2008 ▶); program(s) used to refine structure: *SHELXL97* (Sheldrick, 2008 ▶); molecular graphics: *ORTEP-3 for Windows* (Farrugia, 1997 ▶); software used to prepare material for publication: *WinGX* (Farrugia, 1999 ▶).

## Supplementary Material

Crystal structure: contains datablocks I, global. DOI: 10.1107/S1600536809041208/xu2632sup1.cif

Structure factors: contains datablocks I. DOI: 10.1107/S1600536809041208/xu2632Isup2.hkl

Additional supplementary materials:  crystallographic information; 3D view; checkCIF report

## Figures and Tables

**Table 1:** Selected bond lengths (Å)

Zn1—O1	2.051 (2)
Zn1—O2	2.296 (2)
Zn1—O3	1.9394 (17)
Zn1—N1	2.0569 (18)
Zn1—N2	2.0850 (18)

**Table 2:** Hydrogen-bond geometry (Å, °)

*D*—H⋯*A*	*D*—H	H⋯*A*	*D*⋯*A*	*D*—H⋯*A*
N3—H3*A*⋯O4^i^	0.86	2.21	3.011 (3)	154
N3—H3*B*⋯O4^ii^	0.86	2.03	2.854 (3)	160
N4—H4*A*⋯O5^iii^	0.86	2.02	2.848 (3)	162
N5—H5*A*⋯O5^iv^	0.86	2.40	3.151 (3)	147
N6—H6*A*⋯O7^v^	0.86	2.22	3.047 (4)	162
O7—H71⋯O6^v^	0.88 (4)	1.94 (4)	2.799 (3)	165 (4)
O7—H72⋯O1	0.88 (5)	1.99 (6)	2.828 (4)	157 (7)
C15—H15⋯O6^vi^	0.93	2.45	3.332 (3)	157
C16—H16⋯O4^ii^	0.93	2.53	3.426 (3)	162

Symmetry codes: (i) 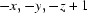; (ii) 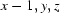; (iii) 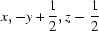; (iv) 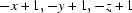; (v) 
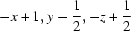
; (vi) 
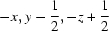
.

**Fig. 1.**
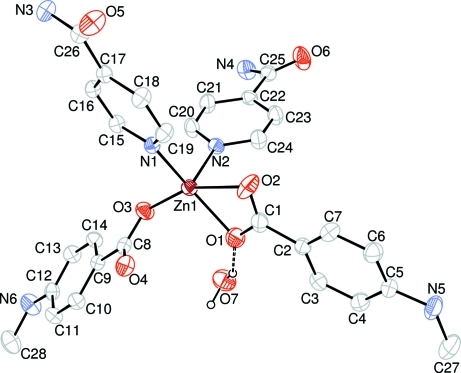
The molecular structure of the title molecule with the atom-numbering scheme. Displacement ellipsoids are drawn at the 30% probability level. Dashed line indicates the hydrogen-bonding.

## supplementary crystallographic information

### Comment

As a part of our ongoing investigation on transition metal complexes of nicotinamide (NA), one form of niacin (Krishnamachari, 1974), and/or the nicotinic acid derivative *N*,*N*-diethylnicotinamide (DENA), an important respiratory stimulant (Bigoli *et al.*, 1972), the title compound was synthesized and its crystal structure is reported herein.

The title compound, (I), is a monomeric complex, where the Zn^II^ ion is surrounded by two methylaminobenzoate (MAB) and two isonicotinamide (INA) ligands. One of the MAB ions acts as a bidentate ligand, while the other MAB and two INA are monodentate ligands. The structures of similar complexes of Zn^II^ ion, [Zn_2_(C_10_H_14_N_2_O)_2_(C_7_H_5_O_3_)_4_].2H_2_O, (II) (Hökelek & Necefoğlu, 1996) and [Zn(C_9_H_10_NO_2_)_2_(C_6_H_6_N_2_O)(H_2_O)_2_], (III) (Hökelek *et al.*, 2009) have also been determined.

In the title compound (Fig. 1), the average Zn—O bond length (Table 1) is 2.095 (2) Å and the Zn atom is displaced out of the least-squares planes of the carboxylate groups (O1/C1/O2) and (O3/C8/O4) by 0.0717 (3) Å and 0.2813 (3) Å, respectively. The dihedral angle between the planar carboxylate groups and the adjacent benzene rings A (C2—C7) and B (C9—C14) are 8.52 (22)° and 5.10 (14)°, respectively, while those between rings A, B, C (N1/C15—C19) and D (N2/C20—C24) are A/B = 71.82 (7), A/C = 14.44 (6), A/D = 84.05 (8), B/C = 72.21 (7), B/D = 47.00 (8) and C/D = 74.92 (7) °. The intramolecular O—H···O hydrogen bond (Table 2) links the water molecule to the carboxylate group (O1/C1/O2). In (I), the O1—Zn1—O2 angle is 59.02 (8)°. The corresponding O—M—O (where *M* is a metal) angles are 58.3 (3)° in (II), 60.03 (6)° in (III) and 55.2 (1)° in [Cu(Asp)_2_(py)_2_] (where Asp is acetylsalicylate and py is pyridine) [(IV); Greenaway *et al.*, 1984].

In the crystal structure, intramolecular O—H···O and intermolecular O—H···O, N—H···O and C—H···O hydrogen bonds (Table 2) link the molecules into a supramolecular structure, in which they may be effective in the stabilization of the structure.

### Experimental

The title compound was prepared by the reaction of ZnSO_4_.H_2_O (0.9 g, 5 mmol) in H_2_O (30 ml) and INA (1.22 g, 10 mmol) in H_2_O (20 ml) with sodium 4-methylaminobenzoate (1.74 g, 10 mmol) in H_2_O (50 ml). The mixture was filtered and set aside to crystallize at ambient temperature for one week, giving yellow single crystals.

### Refinement

Atoms H71 and H72 (for H_2_O) were located in difference Fourier map and refined isotropically, with restrains of O7—H71 = 0.88 (4), O7—H72 = 0.88 (5) Å and H71—O7—H72 = 106 (4)°. The remaining H atoms were positioned geometrically with N—H = 0.86 Å (for NH and NH_2_) and C—H = 0.93 and 0.96 Å for aromatic and methyl H atoms and constrained to ride on their parent atoms, with *U*_iso_(H) = *xU*_eq_(C,N), where *x* = 1.5 for methyl H and *x* = 1.2 for all other H atoms.

### Crystal data

**Table:**

[Zn(C_8_H_8_NO_2_)_2_(C_6_H_6_N_2_O)_2_]·H_2_O	*F*(000) = 1304
*M**_r_* = 627.95	*D*_x_ = 1.437 Mg m^−^^3^
Monoclinic, *P*2_1_/*c*	Mo *K*α radiation, λ = 0.71073 Å
Hall symbol: -P 2ybc	Cell parameters from 6603 reflections
*a* = 8.1323 (8) Å	θ = 2.7–25.3°
*b* = 13.2098 (12) Å	µ = 0.90 mm^−^^1^
*c* = 27.219 (3) Å	*T* = 294 K
β = 96.949 (6)°	Block, yellow
*V* = 2902.6 (5) Å^3^	0.55 × 0.45 × 0.35 mm
*Z* = 4	

### Data collection

**Table:**

Bruker Kappa APEXII CCD area-detector diffractometer	7249 independent reflections
Radiation source: fine-focus sealed tube	5243 reflections with *I* > 2σ(*I*)
graphite	*R*_int_ = 0.047
φ and ω scans	θ_max_ = 28.5°, θ_min_ = 2.2°
Absorption correction: multi-scan (*SADABS*; Bruker, 2005)	*h* = −10→10
*T*_min_ = 0.618, *T*_max_ = 0.725	*k* = −17→17
27836 measured reflections	*l* = −27→36

### Refinement

**Table:**

Refinement on *F*^2^	Primary atom site location: structure-invariant direct methods
Least-squares matrix: full	Secondary atom site location: difference Fourier map
*R*[*F*^2^ > 2σ(*F*^2^)] = 0.042	Hydrogen site location: inferred from neighbouring sites
*wR*(*F*^2^) = 0.110	H atoms treated by a mixture of independent and constrained refinement
*S* = 1.02	*w* = 1/[σ^2^(*F*_o_^2^) + (0.046*P*)^2^ + 0.981*P*] where *P* = (*F*_o_^2^ + 2*F*_c_^2^)/3
7249 reflections	(Δ/σ)_max_ < 0.001
389 parameters	Δρ_max_ = 0.62 e Å^−^^3^
3 restraints	Δρ_min_ = −0.32 e Å^−^^3^

### Special details

**Table:**

Geometry. All e.s.d.'s (except the e.s.d. in the dihedral angle between two l.s. planes) are estimated using the full covariance matrix. The cell e.s.d.'s are taken into account individually in the estimation of e.s.d.'s in distances, angles and torsion angles; correlations between e.s.d.'s in cell parameters are only used when they are defined by crystal symmetry. An approximate (isotropic) treatment of cell e.s.d.'s is used for estimating e.s.d.'s involving l.s. planes.
Refinement. Refinement of *F*^2^ against ALL reflections. The weighted *R*-factor *wR* and goodness of fit *S* are based on *F*^2^, conventional *R*-factors *R* are based on *F*, with *F* set to zero for negative *F*^2^. The threshold expression of *F*^2^ > σ(*F*^2^) is used only for calculating *R*-factors(gt) *etc*. and is not relevant to the choice of reflections for refinement. *R*-factors based on *F*^2^ are statistically about twice as large as those based on *F*, and *R*- factors based on ALL data will be even larger.

### Fractional atomic coordinates and isotropic or equivalent isotropic displacement parameters (Å^2^)

**Table:**

	*x*	*y*	*z*	*U*_iso_*/*U*_eq_	
Zn1	0.38843 (3)	0.14401 (2)	0.382846 (9)	0.03967 (9)	
O1	0.6266 (3)	0.19218 (15)	0.37937 (9)	0.0729 (6)	
O2	0.4834 (3)	0.29273 (19)	0.41966 (8)	0.0767 (6)	
O3	0.3923 (2)	0.00547 (13)	0.35943 (7)	0.0563 (5)	
O4	0.5680 (2)	−0.04606 (13)	0.42345 (6)	0.0530 (4)	
O5	−0.1909 (2)	0.20940 (16)	0.54876 (6)	0.0642 (5)	
O6	0.0275 (3)	0.41634 (17)	0.17505 (9)	0.0875 (7)	
O7	0.7484 (4)	0.0760 (2)	0.30434 (10)	0.0854 (7)	
H71	0.817 (5)	0.028 (3)	0.3165 (17)	0.138 (19)*	
H72	0.739 (10)	0.118 (4)	0.3290 (18)	0.29 (4)*	
N1	0.2088 (2)	0.13652 (13)	0.42983 (6)	0.0379 (4)	
N2	0.2637 (2)	0.20452 (15)	0.31803 (7)	0.0414 (4)	
N3	−0.3353 (3)	0.09359 (16)	0.50163 (7)	0.0474 (5)	
H3A	−0.4175	0.0971	0.5187	0.057*	
H3B	−0.3393	0.0531	0.4768	0.057*	
N4	−0.0387 (3)	0.2613 (2)	0.14760 (8)	0.0648 (6)	
H4A	−0.0868	0.2837	0.1198	0.078*	
H4B	−0.0350	0.1973	0.1534	0.078*	
N5	1.1355 (3)	0.5607 (2)	0.42313 (12)	0.0793 (8)	
H5A	1.1184	0.6159	0.4385	0.095*	
N6	0.5279 (4)	−0.43347 (18)	0.28178 (9)	0.0701 (7)	
H6A	0.4608	−0.4427	0.2552	0.084*	
C1	0.6150 (4)	0.2730 (2)	0.40298 (10)	0.0565 (7)	
C2	0.7569 (3)	0.34465 (18)	0.40946 (9)	0.0465 (6)	
C3	0.8948 (3)	0.3316 (2)	0.38584 (11)	0.0561 (7)	
H3	0.9031	0.2743	0.3664	0.067*	
C4	1.0221 (3)	0.4018 (2)	0.39029 (11)	0.0596 (7)	
H4	1.1143	0.3912	0.3738	0.072*	
C5	1.0136 (3)	0.4877 (2)	0.41909 (11)	0.0535 (6)	
C6	0.8760 (3)	0.4990 (2)	0.44393 (10)	0.0557 (6)	
H6	0.8683	0.5550	0.4642	0.067*	
C7	0.7507 (3)	0.4289 (2)	0.43909 (10)	0.0523 (6)	
H7	0.6596	0.4383	0.4562	0.063*	
C8	0.4882 (3)	−0.06043 (17)	0.38227 (8)	0.0409 (5)	
C9	0.4992 (3)	−0.15862 (16)	0.35668 (8)	0.0379 (5)	
C10	0.6074 (3)	−0.23362 (18)	0.37532 (8)	0.0454 (6)	
H10	0.6740	−0.2226	0.4051	0.055*	
C11	0.6197 (3)	−0.32501 (19)	0.35091 (9)	0.0492 (6)	
H11	0.6943	−0.3740	0.3642	0.059*	
C12	0.5205 (3)	−0.34305 (18)	0.30670 (9)	0.0463 (6)	
C13	0.4099 (3)	−0.2684 (2)	0.28777 (9)	0.0513 (6)	
H13	0.3418	−0.2797	0.2583	0.062*	
C14	0.4007 (3)	−0.17839 (19)	0.31230 (9)	0.0451 (5)	
H14	0.3267	−0.1292	0.2989	0.054*	
C15	0.0795 (3)	0.07424 (17)	0.41887 (8)	0.0393 (5)	
H15	0.0824	0.0285	0.3930	0.047*	
C16	−0.0571 (3)	0.07462 (16)	0.44399 (8)	0.0377 (5)	
H16	−0.1450	0.0308	0.4349	0.045*	
C17	−0.0624 (3)	0.14121 (16)	0.48315 (7)	0.0368 (5)	
C18	0.0745 (3)	0.20295 (19)	0.49524 (9)	0.0474 (6)	
H18	0.0774	0.2471	0.5219	0.057*	
C19	0.2050 (3)	0.19921 (19)	0.46815 (9)	0.0467 (6)	
H19	0.2948	0.2420	0.4767	0.056*	
C20	0.1486 (3)	0.1521 (2)	0.29058 (10)	0.0546 (7)	
H20	0.1208	0.0884	0.3016	0.066*	
C21	0.0679 (3)	0.1870 (2)	0.24650 (10)	0.0562 (7)	
H21	−0.0132	0.1477	0.2286	0.067*	
C22	0.1084 (3)	0.28063 (18)	0.22918 (8)	0.0438 (5)	
C23	0.2258 (4)	0.3356 (2)	0.25815 (10)	0.0563 (7)	
H23	0.2548	0.3998	0.2482	0.068*	
C24	0.3005 (3)	0.2961 (2)	0.30173 (10)	0.0538 (6)	
H24	0.3800	0.3346	0.3208	0.065*	
C25	0.0290 (3)	0.3249 (2)	0.18116 (10)	0.0536 (6)	
C26	−0.2036 (3)	0.15014 (18)	0.51356 (8)	0.0421 (5)	
C27	1.2895 (4)	0.5493 (3)	0.40301 (19)	0.1095 (15)	
H27A	1.3565	0.6084	0.4104	0.164*	
H27B	1.2682	0.5408	0.3678	0.164*	
H27C	1.3469	0.4909	0.4174	0.164*	
C28	0.6406 (5)	−0.5118 (2)	0.29771 (13)	0.0799 (10)	
H28A	0.6176	−0.5701	0.2769	0.120*	
H28B	0.6288	−0.5294	0.3313	0.120*	
H28C	0.7517	−0.4892	0.2957	0.120*	

### Atomic displacement parameters (Å^2^)

**Table:**

	*U*^11^	*U*^22^	*U*^33^	*U*^12^	*U*^13^	*U*^23^
Zn1	0.03984 (15)	0.03946 (15)	0.03854 (14)	0.00064 (12)	−0.00003 (10)	−0.00057 (11)
O1	0.0594 (12)	0.0508 (12)	0.1023 (17)	−0.0087 (10)	−0.0158 (11)	−0.0037 (11)
O2	0.0548 (12)	0.1030 (18)	0.0720 (13)	−0.0330 (12)	0.0069 (11)	0.0000 (12)
O3	0.0581 (11)	0.0415 (9)	0.0678 (12)	0.0058 (8)	0.0019 (9)	−0.0055 (9)
O4	0.0616 (11)	0.0534 (10)	0.0436 (9)	−0.0119 (9)	0.0054 (8)	−0.0142 (8)
O5	0.0619 (12)	0.0890 (15)	0.0424 (9)	−0.0183 (11)	0.0093 (8)	−0.0312 (10)
O6	0.0969 (17)	0.0636 (14)	0.0910 (16)	−0.0088 (12)	−0.0338 (13)	0.0343 (12)
O7	0.0896 (18)	0.0896 (18)	0.0740 (15)	0.0117 (15)	−0.0021 (13)	0.0119 (14)
N1	0.0399 (10)	0.0368 (10)	0.0360 (9)	−0.0024 (8)	0.0005 (8)	−0.0004 (8)
N2	0.0444 (11)	0.0430 (11)	0.0362 (9)	0.0032 (9)	0.0022 (8)	0.0038 (8)
N3	0.0493 (12)	0.0569 (13)	0.0367 (10)	−0.0133 (10)	0.0086 (9)	−0.0088 (9)
N4	0.0757 (16)	0.0712 (16)	0.0428 (12)	0.0031 (13)	−0.0126 (11)	0.0135 (11)
N5	0.0610 (15)	0.0601 (15)	0.120 (2)	−0.0228 (13)	0.0253 (16)	−0.0080 (15)
N6	0.099 (2)	0.0504 (14)	0.0573 (14)	0.0041 (13)	−0.0066 (13)	−0.0171 (11)
C1	0.0561 (16)	0.0532 (16)	0.0541 (15)	−0.0097 (13)	−0.0185 (13)	0.0166 (13)
C2	0.0429 (12)	0.0447 (13)	0.0494 (13)	−0.0023 (11)	−0.0047 (11)	0.0112 (11)
C3	0.0574 (16)	0.0388 (13)	0.0714 (18)	0.0053 (12)	0.0052 (14)	0.0010 (12)
C4	0.0484 (15)	0.0549 (16)	0.0784 (19)	0.0017 (13)	0.0194 (14)	0.0010 (15)
C5	0.0462 (14)	0.0456 (14)	0.0680 (17)	−0.0064 (12)	0.0038 (12)	0.0059 (13)
C6	0.0540 (15)	0.0538 (15)	0.0583 (15)	−0.0052 (13)	0.0026 (13)	−0.0087 (13)
C7	0.0421 (13)	0.0636 (16)	0.0512 (14)	−0.0058 (12)	0.0058 (11)	−0.0014 (13)
C8	0.0411 (12)	0.0401 (12)	0.0434 (12)	−0.0090 (10)	0.0124 (10)	−0.0036 (10)
C9	0.0391 (11)	0.0383 (12)	0.0363 (11)	−0.0043 (10)	0.0049 (9)	−0.0004 (9)
C10	0.0498 (14)	0.0471 (13)	0.0372 (12)	−0.0017 (11)	−0.0037 (10)	−0.0028 (10)
C11	0.0554 (15)	0.0437 (13)	0.0471 (13)	0.0074 (11)	0.0007 (11)	0.0014 (11)
C12	0.0565 (14)	0.0412 (13)	0.0416 (12)	−0.0066 (11)	0.0070 (11)	−0.0027 (10)
C13	0.0555 (15)	0.0549 (15)	0.0408 (12)	−0.0049 (12)	−0.0058 (11)	−0.0071 (11)
C14	0.0478 (13)	0.0459 (13)	0.0400 (12)	0.0020 (11)	−0.0011 (10)	−0.0005 (10)
C15	0.0476 (13)	0.0364 (11)	0.0327 (10)	−0.0043 (10)	0.0000 (9)	−0.0051 (9)
C16	0.0425 (12)	0.0356 (11)	0.0332 (10)	−0.0059 (10)	−0.0027 (9)	−0.0021 (9)
C17	0.0423 (11)	0.0380 (11)	0.0281 (9)	−0.0024 (10)	−0.0034 (8)	0.0026 (9)
C18	0.0518 (14)	0.0511 (14)	0.0380 (11)	−0.0115 (12)	0.0005 (11)	−0.0147 (11)
C19	0.0454 (13)	0.0498 (14)	0.0438 (12)	−0.0127 (11)	0.0016 (11)	−0.0120 (11)
C20	0.0638 (16)	0.0461 (14)	0.0501 (14)	−0.0102 (13)	−0.0092 (12)	0.0121 (12)
C21	0.0577 (16)	0.0540 (15)	0.0519 (14)	−0.0137 (13)	−0.0137 (12)	0.0094 (12)
C22	0.0408 (12)	0.0489 (14)	0.0410 (12)	0.0050 (11)	0.0017 (10)	0.0080 (10)
C23	0.0637 (17)	0.0475 (15)	0.0547 (15)	−0.0075 (13)	−0.0042 (13)	0.0120 (12)
C24	0.0596 (16)	0.0493 (15)	0.0490 (14)	−0.0086 (13)	−0.0081 (12)	0.0054 (12)
C25	0.0438 (13)	0.0593 (16)	0.0553 (15)	0.0013 (12)	−0.0036 (12)	0.0173 (13)
C26	0.0459 (12)	0.0500 (13)	0.0290 (10)	−0.0034 (11)	−0.0005 (9)	−0.0014 (10)
C27	0.061 (2)	0.091 (3)	0.181 (5)	−0.025 (2)	0.035 (3)	0.004 (3)
C28	0.119 (3)	0.0436 (16)	0.078 (2)	0.0090 (18)	0.013 (2)	−0.0122 (15)

### Geometric parameters (Å, °)

**Table:**

Zn1—O1	2.051 (2)	C8—C9	1.480 (3)
Zn1—O2	2.296 (2)	C9—C10	1.380 (3)
Zn1—O3	1.9394 (17)	C9—C14	1.391 (3)
Zn1—N1	2.0569 (18)	C10—H10	0.9300
Zn1—N2	2.0850 (18)	C11—C10	1.388 (3)
O1—C1	1.256 (4)	C11—H11	0.9300
O3—C8	1.279 (3)	C12—N6	1.379 (3)
O5—C26	1.232 (3)	C12—C11	1.386 (3)
O6—C25	1.219 (3)	C13—C12	1.391 (4)
O7—H71	0.88 (4)	C13—H13	0.9300
O7—H72	0.88 (5)	C14—C13	1.370 (3)
N1—C15	1.340 (3)	C14—H14	0.9300
N1—C19	1.335 (3)	C15—H15	0.9300
N2—C20	1.321 (3)	C16—C15	1.373 (3)
N2—C24	1.335 (3)	C16—H16	0.9300
N3—C26	1.314 (3)	C17—C16	1.387 (3)
N3—H3A	0.8600	C17—C26	1.499 (3)
N3—H3B	0.8600	C18—C17	1.387 (3)
N4—H4A	0.8600	C18—H18	0.9300
N4—H4B	0.8600	C19—C18	1.365 (3)
N5—C27	1.434 (4)	C19—H19	0.9300
N5—H5A	0.8600	C20—C21	1.376 (3)
N6—C28	1.415 (4)	C20—H20	0.9300
N6—H6A	0.8600	C21—C22	1.378 (3)
C1—O2	1.240 (4)	C21—H21	0.9300
C1—C2	1.486 (4)	C22—C23	1.371 (3)
C2—C3	1.369 (4)	C22—C25	1.504 (3)
C2—C7	1.378 (4)	C23—C24	1.369 (3)
C3—C4	1.385 (4)	C23—H23	0.9300
C3—H3	0.9300	C24—H24	0.9300
C4—C5	1.386 (4)	C25—N4	1.312 (4)
C4—H4	0.9300	C27—H27A	0.9600
C5—N5	1.377 (3)	C27—H27B	0.9600
C5—C6	1.384 (4)	C27—H27C	0.9600
C6—C7	1.372 (4)	C28—H28A	0.9600
C6—H6	0.9300	C28—H28B	0.9600
C7—H7	0.9300	C28—H28C	0.9600
C8—O4	1.240 (3)		
			
O1—Zn1—O2	59.02 (8)	C9—C10—C11	121.9 (2)
O1—Zn1—N1	142.08 (8)	C9—C10—H10	119.1
O1—Zn1—N2	101.97 (8)	C11—C10—H10	119.1
O3—Zn1—O1	102.97 (8)	C10—C11—H11	120.1
O3—Zn1—O2	158.87 (8)	C12—C11—C10	119.8 (2)
O3—Zn1—N1	101.54 (8)	C12—C11—H11	120.1
O3—Zn1—N2	96.27 (8)	N6—C12—C11	121.4 (2)
O3—Zn1—C1	131.97 (9)	N6—C12—C13	119.8 (2)
N1—Zn1—O2	89.80 (7)	C11—C12—C13	118.8 (2)
N1—Zn1—N2	103.61 (7)	C12—C13—H13	119.8
N1—Zn1—C1	116.91 (9)	C14—C13—C12	120.4 (2)
N2—Zn1—O2	98.32 (8)	C14—C13—H13	119.8
N2—Zn1—C1	101.11 (8)	C9—C14—H14	119.1
C1—O1—Zn1	96.36 (19)	C13—C14—C9	121.8 (2)
C1—O2—Zn1	85.43 (18)	C13—C14—H14	119.1
C8—O3—Zn1	121.05 (16)	N1—C15—C16	123.2 (2)
H71—O7—H72	106 (4)	N1—C15—H15	118.4
C15—N1—Zn1	119.23 (14)	C16—C15—H15	118.4
C19—N1—Zn1	122.76 (15)	C15—C16—C17	119.2 (2)
C19—N1—C15	117.6 (2)	C15—C16—H16	120.4
C20—N2—Zn1	121.09 (16)	C17—C16—H16	120.4
C20—N2—C24	117.3 (2)	C16—C17—C18	117.2 (2)
C24—N2—Zn1	121.55 (17)	C16—C17—C26	124.8 (2)
C26—N3—H3A	120.0	C18—C17—C26	117.97 (19)
C26—N3—H3B	120.0	C17—C18—H18	119.8
H3A—N3—H3B	120.0	C19—C18—C17	120.3 (2)
C25—N4—H4A	120.0	C19—C18—H18	119.8
C25—N4—H4B	120.0	N1—C19—C18	122.5 (2)
H4A—N4—H4B	120.0	N1—C19—H19	118.7
C5—N5—C27	123.3 (3)	C18—C19—H19	118.7
C5—N5—H5A	118.3	N2—C20—C21	123.3 (2)
C27—N5—H5A	118.3	N2—C20—H20	118.4
C12—N6—C28	123.3 (2)	C21—C20—H20	118.4
C12—N6—H6A	118.4	C20—C21—C22	119.4 (2)
C28—N6—H6A	118.4	C20—C21—H21	120.3
O1—C1—Zn1	53.97 (14)	C22—C21—H21	120.3
O1—C1—C2	119.8 (3)	C21—C22—C25	123.5 (2)
O2—C1—Zn1	65.22 (15)	C23—C22—C21	117.2 (2)
O2—C1—O1	119.2 (3)	C23—C22—C25	119.3 (2)
O2—C1—C2	121.0 (3)	C22—C23—H23	119.9
C3—C2—C1	122.2 (3)	C24—C23—C22	120.1 (2)
C3—C2—C7	117.8 (2)	C24—C23—H23	119.9
C7—C2—C1	120.0 (2)	N2—C24—C23	122.7 (2)
C2—C3—C4	121.4 (3)	N2—C24—H24	118.7
C2—C3—H3	119.3	C23—C24—H24	118.7
C4—C3—H3	119.3	O6—C25—N4	122.9 (3)
C3—C4—C5	120.7 (3)	O6—C25—C22	120.0 (3)
C3—C4—H4	119.6	N4—C25—C22	117.1 (2)
C5—C4—H4	119.6	O5—C26—N3	122.9 (2)
N5—C5—C4	122.3 (3)	O5—C26—C17	118.8 (2)
N5—C5—C6	120.2 (3)	N3—C26—C17	118.34 (19)
C6—C5—C4	117.6 (2)	N5—C27—H27A	109.5
C5—C6—H6	119.5	N5—C27—H27B	109.5
C7—C6—C5	121.0 (3)	N5—C27—H27C	109.5
C7—C6—H6	119.5	H27A—C27—H27B	109.5
C2—C7—H7	119.2	H27A—C27—H27C	109.5
C6—C7—C2	121.5 (2)	H27B—C27—H27C	109.5
C6—C7—H7	119.2	N6—C28—H28A	109.5
O3—C8—C9	115.8 (2)	N6—C28—H28B	109.5
O4—C8—O3	123.8 (2)	N6—C28—H28C	109.5
O4—C8—C9	120.3 (2)	H28A—C28—H28B	109.5
C10—C9—C8	121.9 (2)	H28A—C28—H28C	109.5
C10—C9—C14	117.3 (2)	H28B—C28—H28C	109.5
C14—C9—C8	120.8 (2)		
			
O2—Zn1—O1—C1	−1.11 (15)	O2—C1—C2—C7	7.5 (4)
O3—Zn1—O1—C1	−169.11 (16)	C1—C2—C3—C4	176.6 (2)
N1—Zn1—O1—C1	−40.1 (2)	C7—C2—C3—C4	−1.8 (4)
N2—Zn1—O1—C1	91.48 (17)	C1—C2—C7—C6	−176.8 (2)
O1—Zn1—O2—C1	1.12 (15)	C3—C2—C7—C6	1.7 (4)
O3—Zn1—O2—C1	35.3 (3)	C2—C3—C4—C5	0.2 (4)
N1—Zn1—O2—C1	158.38 (16)	C3—C4—C5—N5	−177.9 (3)
N2—Zn1—O2—C1	−97.89 (16)	C3—C4—C5—C6	1.6 (4)
O1—Zn1—O3—C8	60.91 (19)	C4—C5—N5—C27	−7.6 (5)
O2—Zn1—O3—C8	31.3 (3)	C6—C5—N5—C27	173.0 (3)
N1—Zn1—O3—C8	−89.93 (18)	N5—C5—C6—C7	177.8 (3)
N2—Zn1—O3—C8	164.76 (18)	C4—C5—C6—C7	−1.7 (4)
C1—Zn1—O3—C8	53.7 (2)	C5—C6—C7—C2	0.1 (4)
O1—Zn1—N1—C15	−163.70 (16)	O3—C8—C9—C10	175.0 (2)
O1—Zn1—N1—C19	24.0 (2)	O3—C8—C9—C14	−4.4 (3)
O2—Zn1—N1—C15	163.68 (17)	O4—C8—C9—C10	−5.6 (3)
O2—Zn1—N1—C19	−8.64 (19)	O4—C8—C9—C14	175.0 (2)
O3—Zn1—N1—C15	−34.28 (18)	C8—C9—C10—C11	−178.9 (2)
O3—Zn1—N1—C19	153.40 (18)	C14—C9—C10—C11	0.5 (4)
N2—Zn1—N1—C15	65.17 (17)	C8—C9—C14—C13	179.4 (2)
N2—Zn1—N1—C19	−107.15 (19)	C10—C9—C14—C13	0.0 (4)
C1—Zn1—N1—C15	175.36 (16)	C12—C11—C10—C9	−0.5 (4)
C1—Zn1—N1—C19	3.0 (2)	C11—C12—N6—C28	−3.0 (4)
O1—Zn1—N2—C20	139.6 (2)	C13—C12—N6—C28	177.9 (3)
O1—Zn1—N2—C24	−38.2 (2)	N6—C12—C11—C10	−179.2 (2)
O2—Zn1—N2—C20	−160.5 (2)	C13—C12—C11—C10	−0.1 (4)
O2—Zn1—N2—C24	21.8 (2)	C14—C13—C12—N6	179.7 (2)
O3—Zn1—N2—C20	34.9 (2)	C14—C13—C12—C11	0.6 (4)
O3—Zn1—N2—C24	−142.9 (2)	C9—C14—C13—C12	−0.6 (4)
N1—Zn1—N2—C20	−68.7 (2)	C17—C16—C15—N1	−1.0 (3)
N1—Zn1—N2—C24	113.6 (2)	C18—C17—C16—C15	−1.2 (3)
C1—Zn1—N2—C20	169.9 (2)	C26—C17—C16—C15	179.7 (2)
C1—Zn1—N2—C24	−7.9 (2)	C16—C17—C26—O5	177.1 (2)
Zn1—O1—C1—O2	2.0 (3)	C16—C17—C26—N3	−2.4 (3)
Zn1—O1—C1—C2	−176.46 (19)	C18—C17—C26—O5	−2.0 (3)
Zn1—O3—C8—O4	9.7 (3)	C18—C17—C26—N3	178.4 (2)
Zn1—O3—C8—C9	−170.82 (14)	C19—C18—C17—C16	2.1 (3)
Zn1—N1—C15—C16	−170.49 (17)	C19—C18—C17—C26	−178.7 (2)
C19—N1—C15—C16	2.2 (3)	N1—C19—C18—C17	−0.8 (4)
Zn1—N1—C19—C18	171.13 (19)	N2—C20—C21—C22	0.7 (5)
C15—N1—C19—C18	−1.3 (4)	C20—C21—C22—C23	−1.7 (4)
Zn1—N2—C20—C21	−177.2 (2)	C20—C21—C22—C25	179.2 (3)
C24—N2—C20—C21	0.6 (4)	C21—C22—C23—C24	1.5 (4)
Zn1—N2—C24—C23	176.9 (2)	C25—C22—C23—C24	−179.4 (3)
C20—N2—C24—C23	−0.9 (4)	C21—C22—C25—O6	156.3 (3)
O1—C1—O2—Zn1	−1.8 (2)	C21—C22—C25—N4	−22.6 (4)
C2—C1—O2—Zn1	176.7 (2)	C23—C22—C25—O6	−22.7 (4)
O1—C1—C2—C3	7.5 (4)	C23—C22—C25—N4	158.3 (3)
O1—C1—C2—C7	−174.1 (2)	C22—C23—C24—N2	−0.2 (4)
O2—C1—C2—C3	−170.9 (3)		

### Hydrogen-bond geometry (Å, °)

**Table:**

*D*—H···*A*	*D*—H	H···*A*	*D*···*A*	*D*—H···*A*
N3—H3A···O4^i^	0.86	2.21	3.011 (3)	154
N3—H3B···O4^ii^	0.86	2.03	2.854 (3)	160
N4—H4A···O5^iii^	0.86	2.02	2.848 (3)	162
N5—H5A···O5^iv^	0.86	2.40	3.151 (3)	147
N6—H6A···O7^v^	0.86	2.22	3.047 (4)	162
O7—H71···O6^v^	0.88 (4)	1.94 (4)	2.799 (3)	165 (4)
O7—H72···O1	0.88 (5)	1.99 (6)	2.828 (4)	157 (7)
C15—H15···O6^vi^	0.93	2.45	3.332 (3)	157
C16—H16···O4^ii^	0.93	2.53	3.426 (3)	162

Symmetry codes: (i) −*x*, −*y*, −*z*+1; (ii) *x*−1, *y*, *z*; (iii) *x*, −*y*+1/2, *z*−1/2; (iv) −*x*+1, −*y*+1, −*z*+1; (v) −*x*+1, *y*−1/2, −*z*+1/2; (vi) −*x*, *y*−1/2, −*z*+1/2.
